# A patient with COVID-19 and bleeding complications due to neurofibromatosis type 1 during VV-ECMO: A case report

**DOI:** 10.1097/MD.0000000000028094

**Published:** 2021-12-23

**Authors:** Keiichiro Shimoyama, Kazunari Azuma, Jun Oda

**Affiliations:** Department of Emergency and Critical Care Medicine, Tokyo Medical University, 6-7-1, Nisi-Shinjuku, Shinjuku-Ku, Japan.

**Keywords:** bleeding, complications, coronavirus disease 2019, extracorporeal membrane oxygenation, neurofibromatosis, von Recklinghausen's disease

## Abstract

**Rationale:**

The many deaths from coronavirus disease (COVID-19) since 2019 have caused global concern. Effective treatment has not yet been established; supportive care is the main treatment. It has been suggested that veno-venous extracorporeal membrane oxygenation (VV-ECMO) may be effective in severe cases that do not respond to ventilator management.

**Patient concerns and diagnosis:**

We report the case of a 68-year-old woman with severe respiratory failure due to COVID-19 who was treated with VV-ECMO but suffered from bleeding complications. She presented with multiple café-au-lait lesions and neurofibromas on her skin and was diagnosed pathologically as having neurofibromatosis type 1(NF1).

**Interventions and outcomes:**

Although she received appropriate anticoagulation therapy with heparin at the initiation of VV-ECMO, she had 5 episodes of severe bleeding, each requiring transcatheter arterial embolization and massive transfusion. In patients with NF1, vascular fragility has been noted due to vascular infiltration of neurofibromas and degeneration of vascular structures. Therefore, the causes of frequent bleeding complications may be related to the fragility of blood vessels in patients with NF1. VV-ECMO in patients with NF1 is likely to result in frequent bleeding complications and the need for massive transfusion.

**Lesson:**

We propose non-anticoagulation treatment strategy for the management of VV-ECMO in patients with NF1. Especially under the COVID-19 pandemic, more careful consideration should be given to the indications for VV-ECMO in patients with NF1.

## 1 Introduction

Coronavirus Disease 2019 (COVID-19) was first reported at the end of 2019 and has spread worldwide causing many deaths. Effective treatment has not yet been established; supportive care is the main therapy. COVID-19 can lead to respiratory failure requiring ventilator management. In severe respiratory failure, patients who fail to respond to intensive therapies may be difficult to save. In these severe cases, veno-venous extracorporeal membrane oxygenation (VV-ECMO) has emerged as a supportive therapy of last resort.^[1]^ The incidence of complications during ECMO management is high, with bleeding being the most common.^[2]^ Although there have been a few successful cases, the efficacy of VV-ECMO in severe respiratory failure, including COVID-19, is controversial; it is not clear in which patients VV-ECMO should be implemented.^[3]^ Risk assessment for hemorrhagic complications is important in the management of VV-ECMO; its indications need to be carefully considered.

We present a case of neurofibromatosis type 1 (NF1) who had frequent bleeding complications during VV-ECMO treatment, requiring multiple transcatheter arterial embolizations (TAE). Ethics approval was waived for this case report. Consent for publication was obtained from the patient. Personal information is anonymized.

## 2 Case presentation

### 2.1 Patient information

A 68-year-old woman was transferred to our hospital with worsening respiratory failure related to COVID-19. She was 9 days from diagnosis of COVID-19 and had been treated with favipiravir and dexamethasone at the previous hospital. She had a history of hypertension and hyperlipidemia.

### 2.2 Clinical findings and clinical course

**Figure 1:**
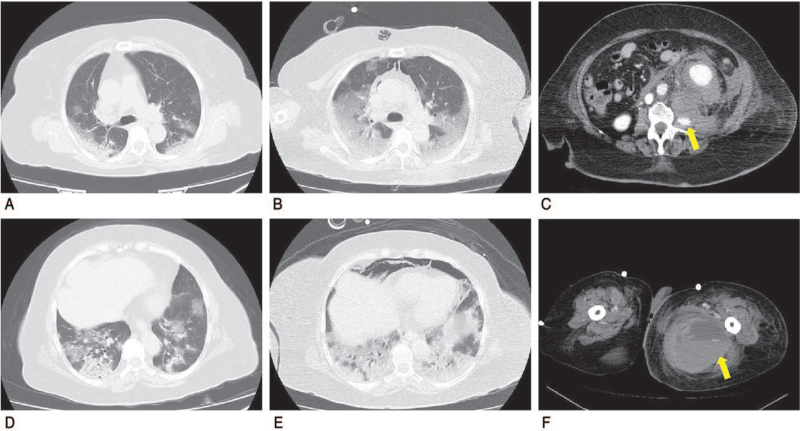
Computed tomography (CT) findings at the bifurcation and the lung base, and contrast-enhanced CT findings. (A) Chest CT performed on day 1 showed extensive ground-glass opacity (GGO). (B) Chest CT performed on day 6 showed further enlargement of the GGO. (C) The contrast-enhanced CT venous phase image obtained on day 13 after admission revealed active bleeding with extravasation of the left retroperitoneal space (arrow). Transcatheter arterial embolization (TAE) was performed using primary embolization material for the L2–L3 left lumbar artery. (D) The contrast-enhanced CT venous phase image obtained on day 28 after admission revealed active bleeding with extravasation in the left thigh muscle (arrow). TAE was immediately performed using primary embolization material for the left deep femoral artery, but extravasation was not clear on femoral artery angiography.

The patient was obese with a height of 140 cm and a body mass index of 36.5. In the emergency department, she had clear consciousness and her vital signs and were as follows: blood pressure, 127/86 mm Hg; heart rate, 77 beats/min; respiratory rate, 28/min; saturation on pulse oximetry, 98% in O_2_ mask 10 L. Because she had dyspnea, she was intubated, placed on a ventilator, and admitted to the emergency intensive care unit (EICU). Favipiravir was terminated and dexamethasone was administered for 10 days. Chest computed tomography (CT) scans at the time of admission are shown in Figure 1A. After intubation, the ventilator settings were: assist control pressure control ventilation; fraction of inspired oxygen, 0.4; positive end-expiratory pressure, 10 cmH_2_O, pressure above positive end-expiratory pressure, 15 cmH_2_O, and partial pressure of arterial oxygen/fraction of inspiratory oxygen ratio (P/F ratio), 174—indicating moderate acute respiratory distress syndrome. It was assumed that the transpulmonary pressure was not large, based on occlusion pressure and physical findings; the patient was maintained under spontaneous breathing. A tracheostomy was performed on day 5 after EICU admission. However, on day 6, there was a sudden increase in respiratory effort and the P/F ratio worsened to 100. Chest CT scans at this time are shown in Figure 1B. The patient was treated in the prone position with muscle relaxants and efforts were made to achieve lung protective ventilation; but, progressive hypoxemia was observed, with a P/F ratio of 72. Since the patient was not old, and Activity of Daily Living was Independent, the indications for VV-ECMO were considered through discussion among a multidisciplinary team that included respiratory physicians, infectious disease specialists, intensivists, and nurses. Pulmonary injuries were thought to be reversible due to the short duration of ventilator management. On day 7 after admission, VV-ECMO was initiated, as follows: right femoral vein drainage, 23Fr HLS cannula (MAQUET Cardiopulmonary, Rastatt, Germany); right internal jugular vein return, 19Fr HLS cannula (MAQUET); centrifugal pump, CAPIOX SP-200 (TERUMO, Tokyo, Japan) (extracorporeal membrane oxygenation [ECMO] flow, 3.4 L; 1430 rpm; delivered fractional oxygen percentage, 1.0; sweep gas, 3 L).

### 2.3 Bleeding complications during ECMO management

**Figure 2:**
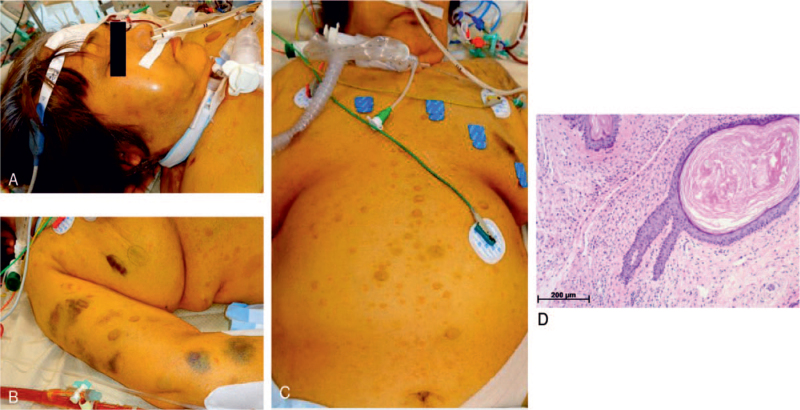
Physical and pathological findings. There was internal bleeding on the buccal mucosa and extremities (A, B) and no external cause was found. Pathological findings of the skin are shown. (C) There were multiple café-au-lait spots and neurofibromas all over the body. (D) Neurofibromas were pathologically confirmed. Pathological findings of skin biopsy showed nodular lesions in the dermis with spindle-shaped tumor cells with abundant nuclear chromatin.

**Figure 3:**
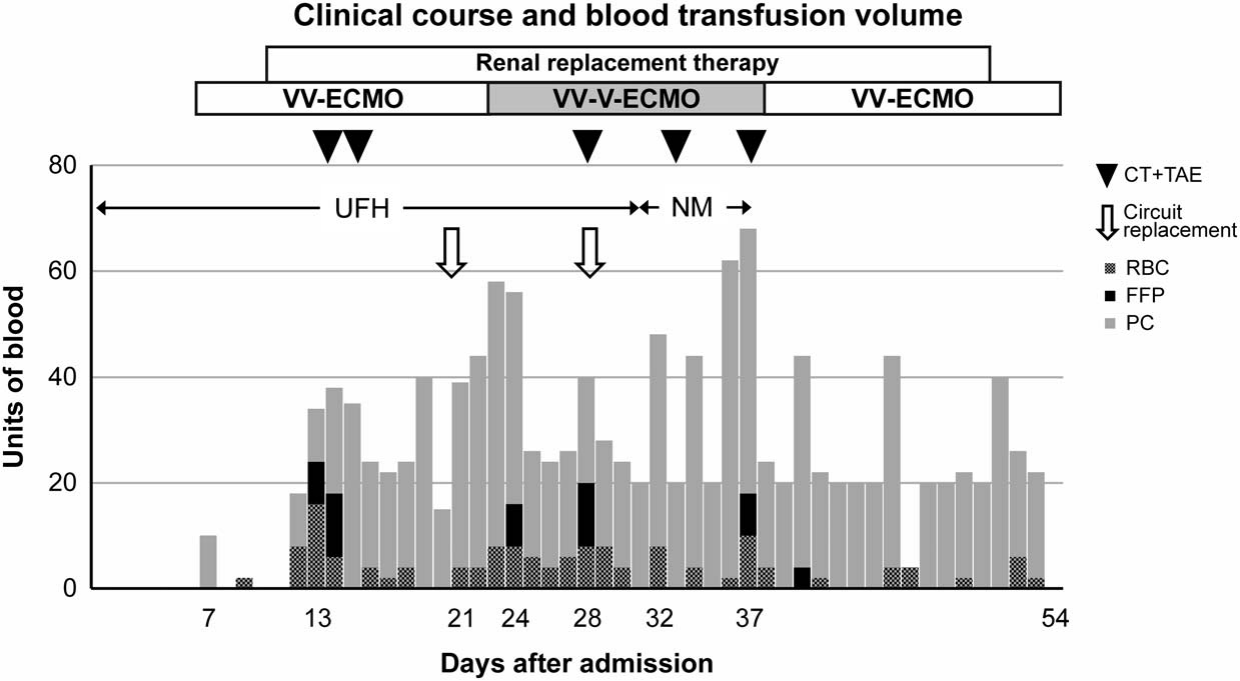
Clinical course and blood transfusion volume. During the patient's course, there were 5 fatal bleeding complications, each of which was treated with transcatheter arterial embolization. Due to increased oxygen demand from sepsis, the configuration was changed to VV-V-ECMO during days 24 to 37 after admission. FFP = fresh frozen plasma, NM = nafamostat mesilate, PC = platelet concentration, RBC = red blood cells, UFH = unfractionated heparin, VV-V-ECMO = veno-veno-venous extracorporeal membrane oxygenation.

After initiation of VV-ECMO, fluid balance was regulated using diuretics and the extracorporeal ultrafiltration method. To maintain safe and stable ECMO for a long period, anticoagulation was established using unfractionated heparin to achieve an activated partial thromboplastin time ratio of 1.5. A platelet transfusion was performed to achieve a platelet count of 8 × 10^3^/μL. However, a subcutaneous hematoma appeared in an area unrelated to external factors, such as restraint and repositioning (Fig. 2A, 2B). On day 13 after admission, ECMO flow decreased abruptly to 1.2 L, with a simultaneous decrease in blood pressure to 79/48 mm Hg. With bleeding complications suspected, a contrast-enhanced CT scan of the trunk was performed. Active bleeding with extravasation of the left retroperitoneal space was confirmed. TAE was immediately performed and ECMO flow and blood pressure were stabilized. (Fig. 1C) Six hours after achieving hemostasis, anticoagulation with unfractionated heparin was restarted. However, on the following day (day 14 after EICU admission), ECMO flow again decreased and hypotension was observed; a bleeding complication was suspected. Contrast-enhanced CT scanning was performed again, and re-bleeding from the same site as previously was confirmed and immediately hemostated using TAE. Similar bleeding complications were observed on days 28, 32, and 37, all of which showed bleeding with extravasation on contrast-enhanced CT scan (Fig. 1D). The bleeding vessels were the lumbar artery (L1–3) and deep femoral artery. Each of these episodes required hemostasis through TAE and massive blood transfusion. Table 1 shows the results of blood collection. No excessive anticoagulation was given. Increasing the platelet level did not stop the bleeding. The slight increase in thrombin-antithrombin complex, elevated fibrinogen degradation products, and platelet decrease in the presence of bleeding complications also suggested a bleeding tendency due to disseminated intravascular coagulation, but the rise in plasmin-α2 plasmin inhibitor complex (PIC) was slight, so it was not considered. The patient was anticoagulated with nafamostat mesilate from day 34, but bleeding complications recurred and anticoagulation was discontinued from day 37, while management continued. After anticoagulation was stopped, no bleeding complications were observed. The clinical course is shown in Figure 3.

**Table 1:** Patients’ laboratory values.

Laboratory value	Day 1	Day 3	Day 7	Day 13	Day 14	Day 28	Day 32	Day 37
WBC (×10 × 3/μL)	13.9	13.2	14.9	26.1	22.5	4.7	2.1	3.5
RBC (×10 × 6/μL)	4.71	4.2	3.75	3.22	3.24	4.1	3.58	3.61
Hb (g/dL)	14.5	12.9	11.6	10.2	9.9	12.7	10.8	11
Plt (×10 × 3/μL)	175	172	53	83	73	79	99	80
T-Bil (mg/dL)	0.47	0.37	0.79	1.02	1.18	16.96	26.6	48.8
D-Bil (mg/dL)	0.14	Unavailable	Unavailable	Unavailable	Unavailable	Unavailable	Unavailable	36.8
AST (U/L)	17	20	61	92	40	234	73	107
ALT (U/L)	21	20	47	250	100	197	232	45
LDH (U/L)	364	356	791	717	462	1766	588	1774
Na (mEq/L)	138	144	142	144	141	138	140	141
K (mEq/L)	4.6	4.7	4.2	4.2	4	5.4	4.3	4.6
Cl (mEq/L)	106	110	106	110	106	105	104	102
BUN (mg/dL)	18.3	37.8	43.5	45.6	44.9	61.3	53.5	62.4
Cre (mg/dL)	0.58	0.51	0.67	0.5	0.53	0.52	0.91	0.74
CRP (mg/dL)	4.7	7.3	27.9	1.1	0.5	10.4	29.3	21.7
KL-6 (U/mL)	Unavailable	383	955	Unavailable	Unavailable	Unavailable	370	Unavailable
APTT (sec)	31.9	47.6	46.1	42.6	44.9	41.3	33	44.2
PT-INR	1.02	1	1.23	1.07	1.08	0.92	0.98	1
Fibrinogen (mg/dL)	565	616	581	299	221	349	496	409
AT III (%)	102.8	104.2	65.7	104	89	96.3	85.4	83
FDP (μg/mL)	3.5	3.6	Unavailable	17.2	10.5	15.2	23.3	28.9
D-dimer (μg/mL)	1.23	1.52	49.8	7.66	5.07	7.25	10.11	12.16
TAT (ng/ml)	Unavailable	3.35	6.15	4.03	Unavailable	15.3	9.98	8.55
PIC (μg/mL)	Unavailable	1.3	5.49	0.81	Unavailable	0.59	1.52	2.32

^∗^Day 3 laboratory values before ECMO; Day 7–37 are laboratory values just before bleeding complications. ALT = alanine aminotransferase, APTT = activated partial thromboplastin time, AST = asparate aminotransferase, ATIII = antithrombin 3, BUN = blood urea nitrogen, Cre = creatinine, D-Bil = direct bilirubin, ECMO = extracorporeal membrane oxygenation, FDP = fibrin/fibrinogen degradation products, Hb = hemoglobin, KL-6 = sialylated carbohydrate antigen, LDH = lactic acid dehydrogenase, PIC = plasmin-α2 plasmin inhibitor complex, Plt = platelet, PT-INR = prothrombin time international normalized ratio, RBC = red blood cells, TAT = thrombin-antithrombin complex, T-Bil = total bilirubin, WBC = white blood cells.

### 2.4 Diagnosis of NF1 and outcome

The patient presented with multiple café-au-lait lesions and neurofibromas over her skin (Fig. 2C). NF1 was suspected; and confirmed by skin biopsy (Fig. 2D). On day 21, bacteremia caused by *Serratia* was found and antimicrobial therapy was required. On day 54, methicillin-resistant *Staphylococcus aureus* bacteremia persisted. On day 57 after admission, the patient was discharged dead from multiple organ failure.

## 3 Discussion

We introduced VV-ECMO for a patient with severe COVID-19, and dealt with multiple life-threatening bleeding complications. We propose no anticoagulation for the management of VV-ECMO in patients with NF1. The indications for VV-ECMO should be considered carefully.

Also known as von Recklinghausen's disease, NF1 is an autosomal dominant genetic disorder in which dysregulation of Ras protein causes tumor growth in various organs. It is reported to be present in 1 in 2500 to 3000 people.^[4]^ Although hemorrhage and embolism due to vasculopathy is reported to be the second leading cause of death in patients with NF1,^[4]^ there is no mention of bleeding in reviews or guidelines, and recognition of bleeding susceptibility in NF1 is not high. However, cases of spontaneous, life-threatening bleeding from large arteries have been reported.^[5]^ Belcastro et al reported bleeding due to weakness of the internal jugular vein, and Hinsch et al reported massive bleeding due to weakness of the vein from the lower abdomen to the pelvic region, which support the possibility of arteriovenous and venous bleeding in NF1.^[6,7]^ This bleeding susceptibility has been attributed to the effect of neurofibromas surrounding arteries, and their degeneration due to intimal proliferation, thinning of the muscle layer, and fragmentation of the elastic layer.^[7]^ Neurofibromas have invaded both arteries and veins.^[5]^ These mechanisms may impart fragility to blood vessels in NF1 patients.

This is the first report of using VV-ECMO in a patient with NF1. Although advances in ECMO have made thrombotic complications less likely, anticoagulation is usually required. Excessive anticoagulation can contribute to bleeding complications, with other factors, such as hyperfibrinolysis and acquired von Willebrand syndrome. In this case, PIC and ADAMTS13 enzyme levels were normal. It is possible that NF1-induced vascular fragility and anticoagulant therapy may have contributed to bleeding, rather than a secondary bleeding tendency caused by ECMO. Since this patient had multiple life-threatening bleeding complications despite appropriate anticoagulation, a strategy of avoiding anticoagulation may be effective when implementing extracorporeal circulation such as ECMO in patients with NF1. Since our patient had 5 life-threatening bleeding episodes requiring hemostasis using TAE and massive blood transfusion, significant medical resources and effort were required. Bleeding complications during ECMO, and blood transfusions, have been reported correlate with increased use of medical resources and in-hospital mortality.^[8]^ The implementation of ECMO in patients with NF1 is likely to lead to bleeding complications requiring blood transfusion, often leading to poor outcomes. When initiating ECMO, the impact on intensive care unit operations and the care of other critically ill patients should be considered.^[3]^ The indications for ECMO in patients with NF1 need to be considered carefully.

## 4 Conclusion

The implementation of VV-ECMO in patients with NF1 may lead to bleeding complications. The indication of VV-ECMO should be considered carefully because of the possibility of overloading intensive care units. If the benefits of VV-ECMO are considered significant for patients with NF1, a non-anticoagulation strategy may be effective.

This is a Japanese case report. Racial differences should be considered in generalizing these findings. More trials of VV-ECMO in patients with NF1 are needed.

## Acknowledgments

The authors would like to thank Mana Sawahata, Kazuma Kimura, Yuki Nishiyama, Daichi Mitsui, Naoki Motohasi, Tsubasa Fujikawa, Masako Sakurai, Kenta Aida, Yuri Ishii, and Syoji Suzuki for treating the patient and lending support.

The authors would also like to thank Enago (www.enago.jp) for the English language review.

## Author contributions

**Conceptualization:** Keiichiro Shimoyama, Jun Oda.

**Formal analysis:** Keiichiro Shimoyama.

**Supervision:** Kazunari Azuma, Jun Oda.

**Writing – original draft:** Keiichiro Shimoyama.

**Writing – review & editing:** Keiichiro Shimoyama.
